# Supplementary material for: Equivalent change enrichment analysis: assessing equivalent and inverse change in biological pathways between diverse experiments
Source: BMC Genomics. 2020 Feb 24;21:180. doi: 10.1186/s12864-020-6589-x (PMC7041296; doi:10.1186/s12864-020-6589-x)
Supplement: Supplementary file 1 — Additional file 1: Supplemental Material: contains additional figures showing the results of the simulation when using varying probabilities of equivalent or inverse change. [file 12864_2020_6589_MOESM1_ESM.pdf]

Supplemental Material for Equivalent Change Enrichment Analysis: Assessing Equivalent and Inverse Change in Biological Pathways between Diverse Experiments

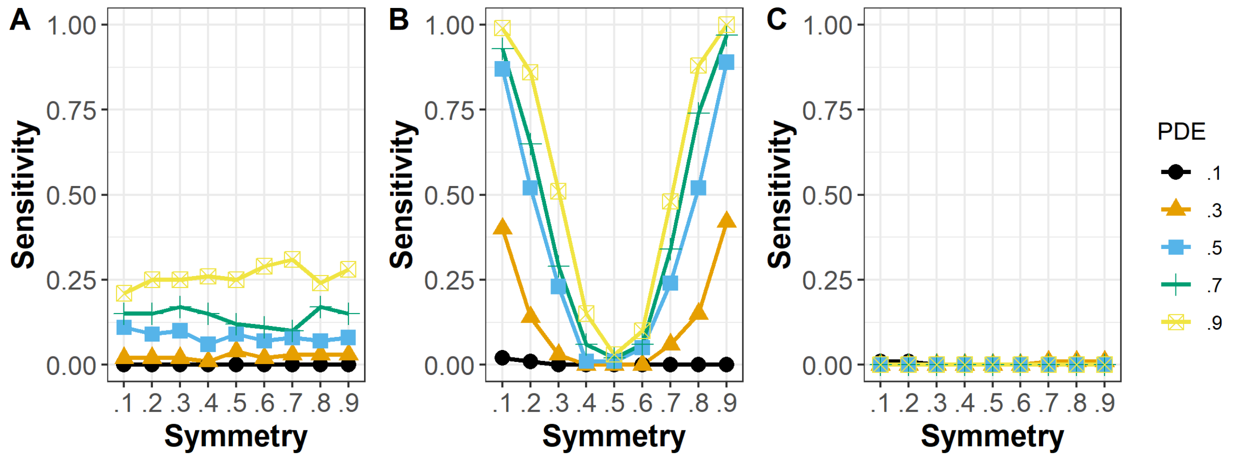

Figure S1: The proportion of equivalently changed pathways detected by each method, when the probability of equivalent change was 0.1. The results are shown for (A) ECEA, (B) GSEA, and (C) ORA.

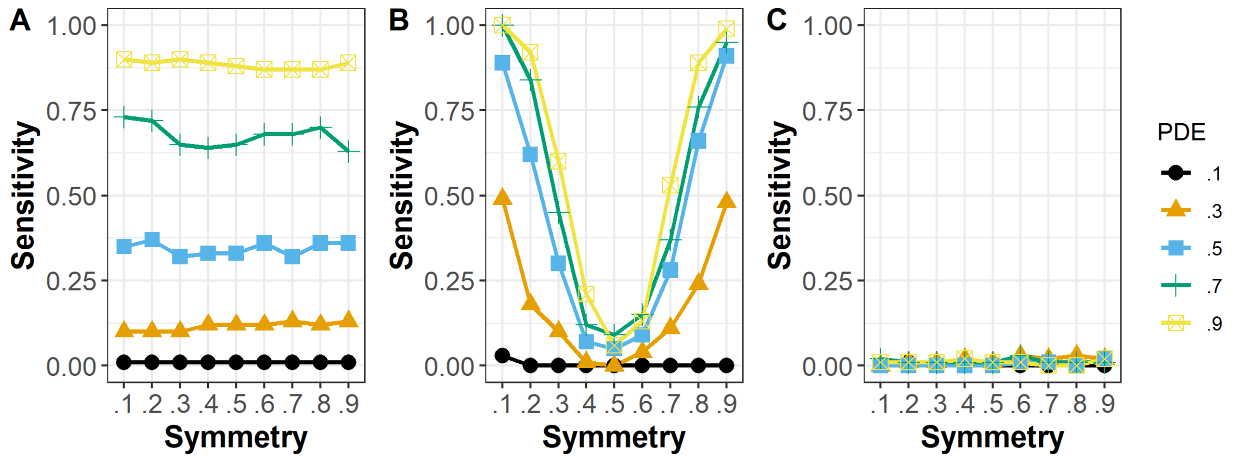

Figure S2: The proportion of equivalently changed pathways detected by each method, when the probability of equivalent change was 0.2. The results are shown for (A) ECEA, (B) GSEA, and (C) ORA.

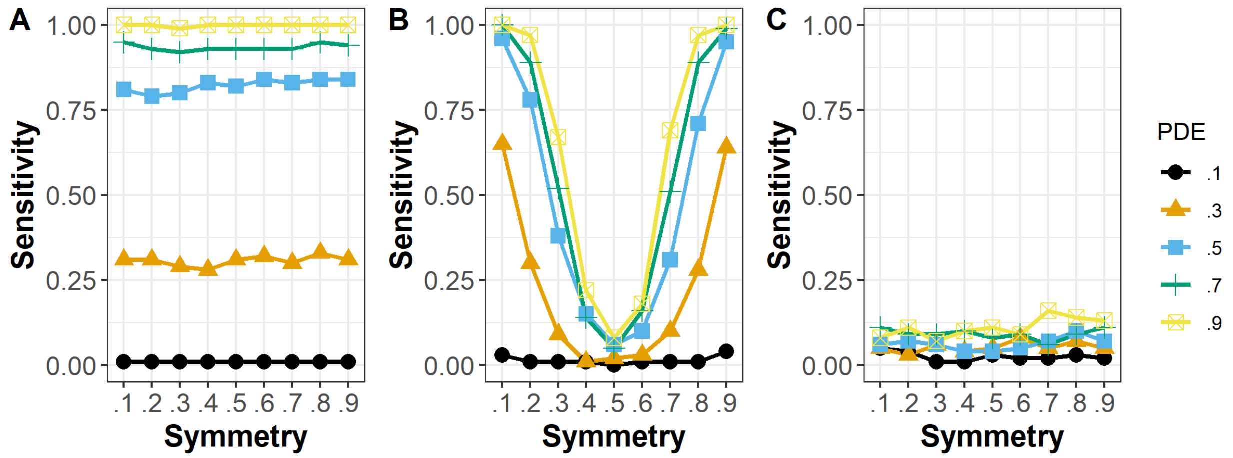

Figure S3: The proportion of equivalently changed pathways detected by each method, when the probability of equivalent change was 0.3. The results are shown for (A) ECEA, (B) GSEA, and (C) ORA.

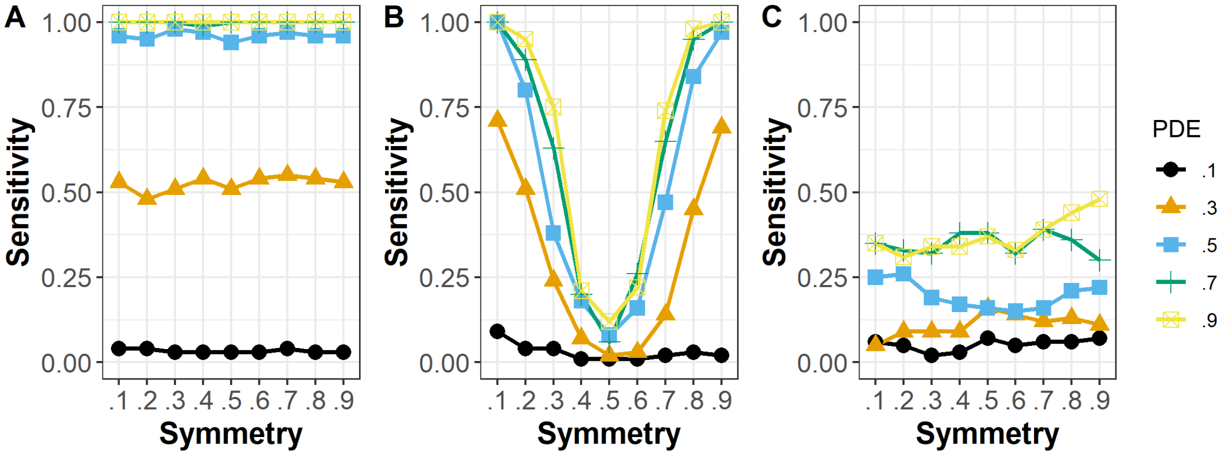

Figure S4: The proportion of equivalently changed pathways detected by each method, when the probability of equivalent change was 0.4. The results are shown for (A) ECEA, (B) GSEA, and (C) ORA.

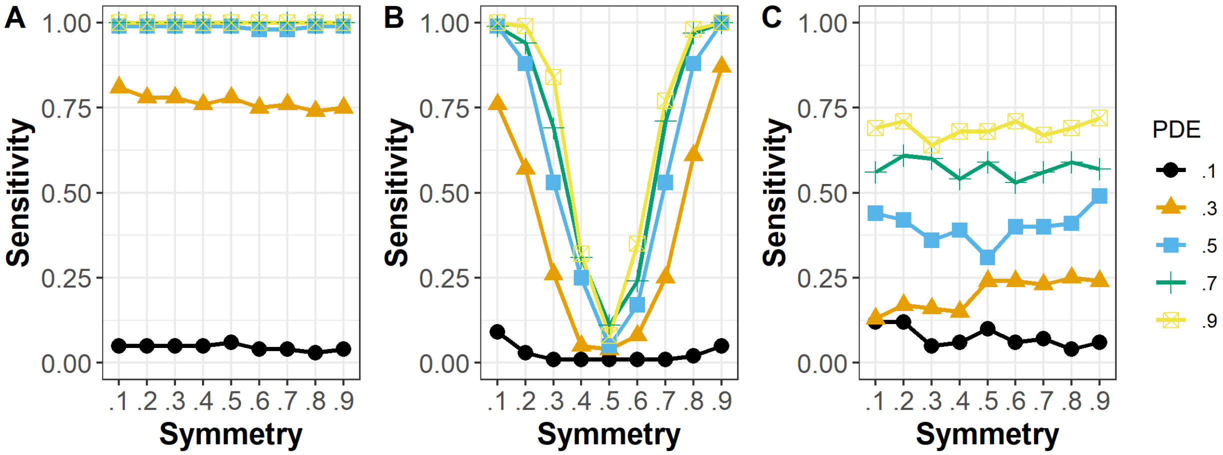

Figure S5: The proportion of equivalently changed pathways detected by each method, when the probability of equivalent change was 0.5. The results are shown for (A) ECEA, (B) GSEA, and (C) ORA.

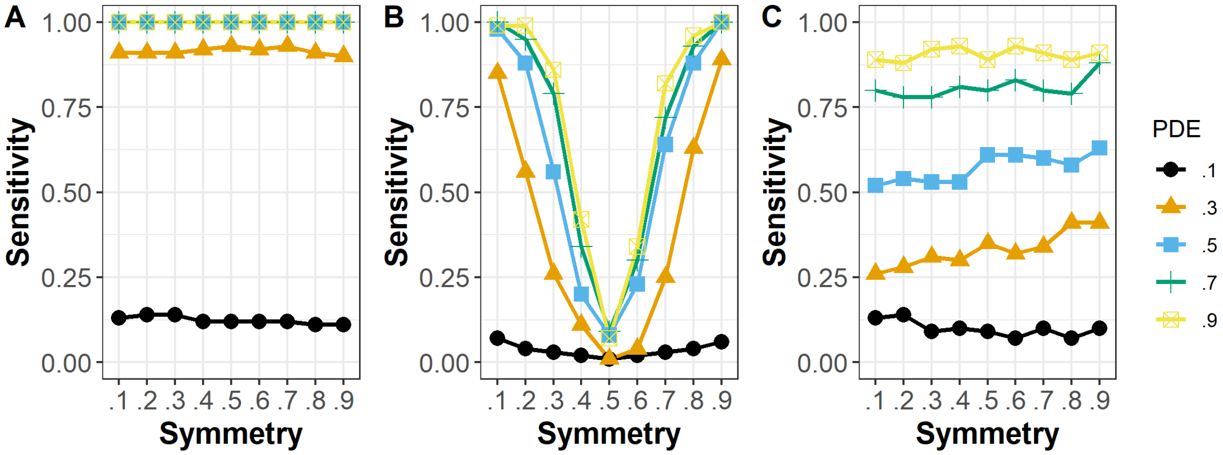

Figure S6: The proportion of equivalently changed pathways detected by each method, when the probability of equivalent change was 0.6. The results are shown for (A) ECEA, (B) GSEA, and (C) ORA.

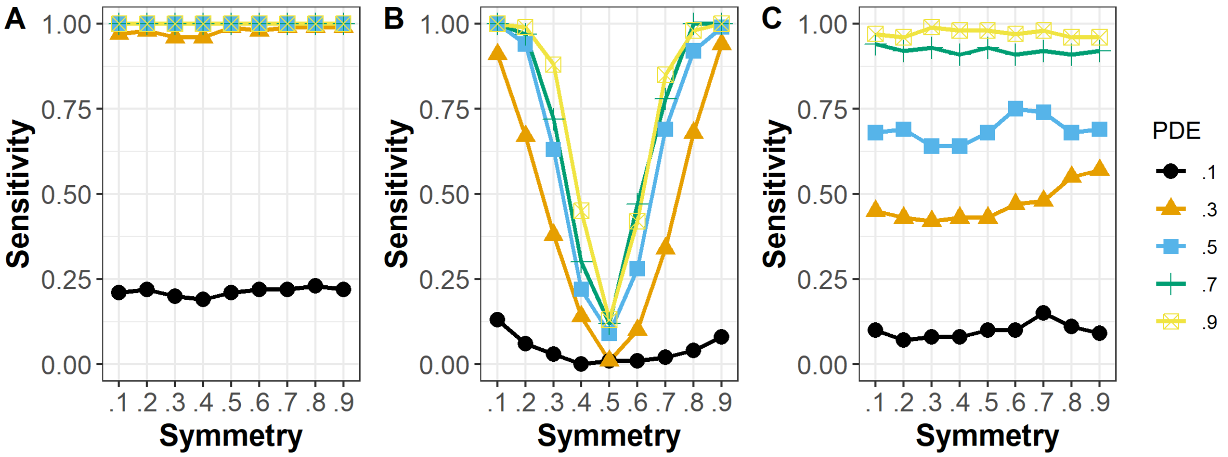

Figure S7: The proportion of equivalently changed pathways detected by each method, when the probability of equivalent change was 0.7. The results are shown for (A) ECEA, (B) GSEA, and (C) ORA.

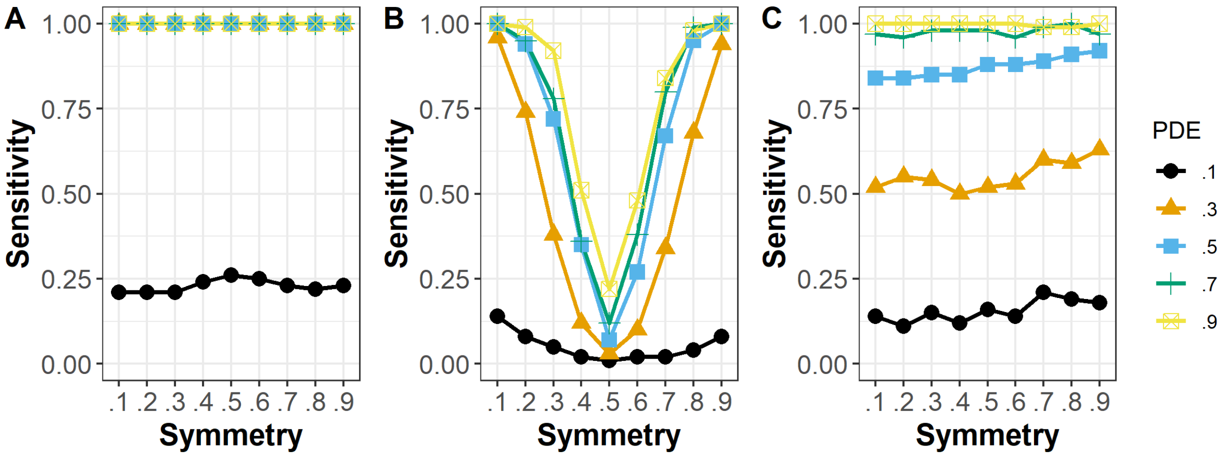

Figure S8: The proportion of equivalently changed pathways detected by each method, when the probability of equivalent change was 0.8. The results are shown for (A) ECEA, (B) GSEA, and (C) ORA.

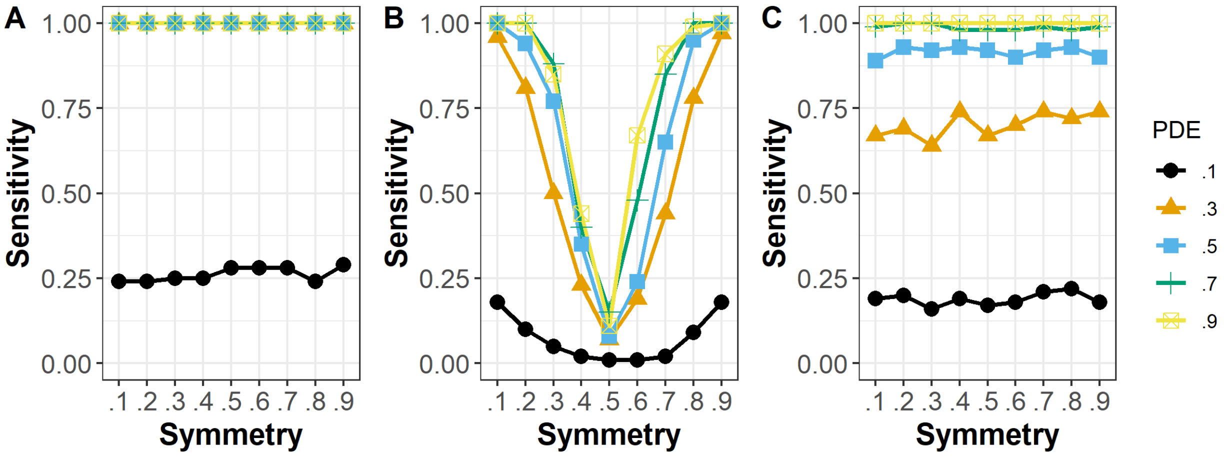

Figure S9: The proportion of equivalently changed pathways detected by each method, when the probability of equivalent change was 0.9. The results are shown for (A) ECEA, (B) GSEA, and (C) ORA.

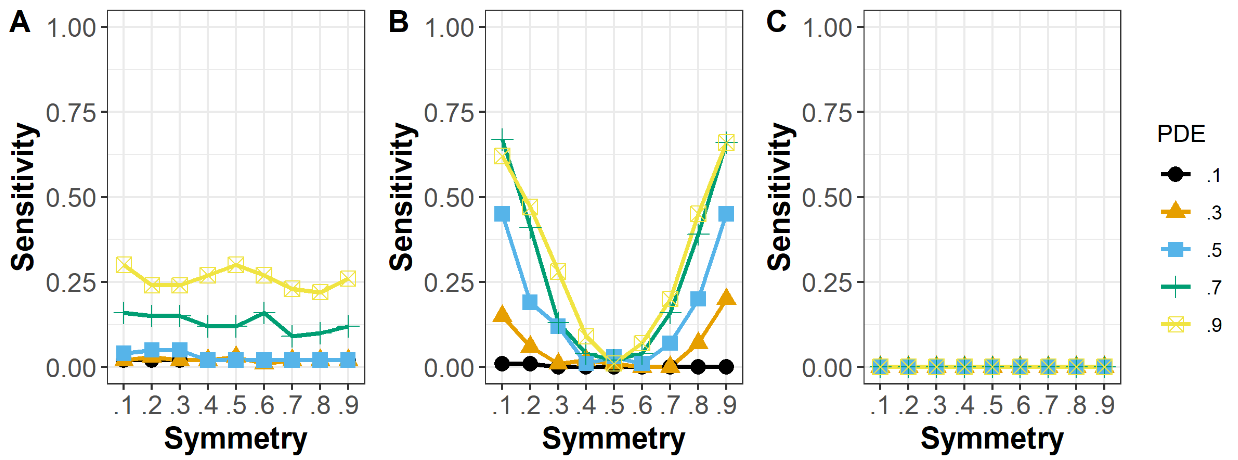

Figure S20: The proportion of inversely changed pathways detected by each method, when the probability of inverse change was 0.1. The results are shown for (A) ECEA, (B) GSEA, and (C) ORA.

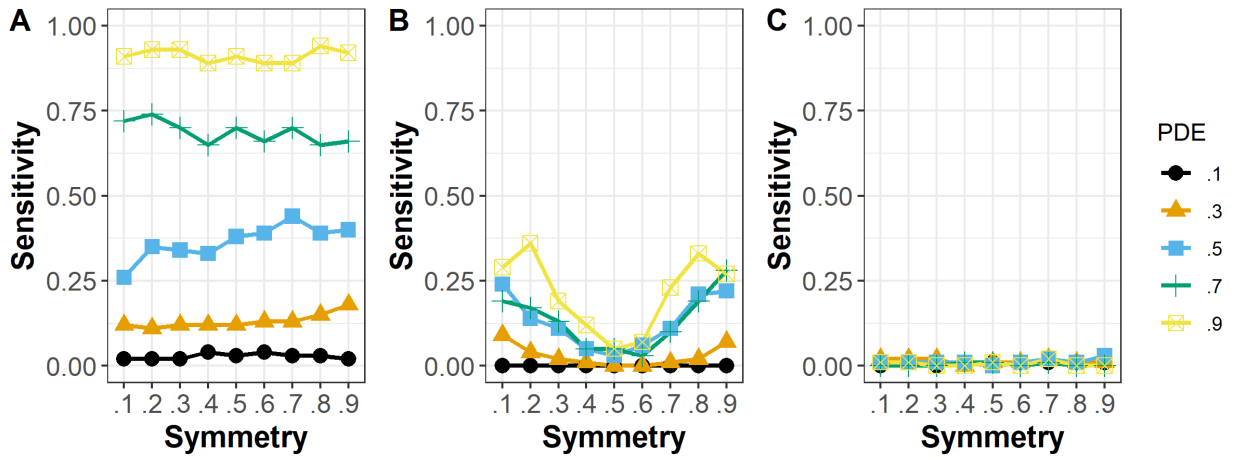

Figure S31: The proportion of inversely changed pathways detected by each method, when the probability of inverse change was 0.2. The results are shown for (A) ECEA, (B) GSEA, and (C) ORA.

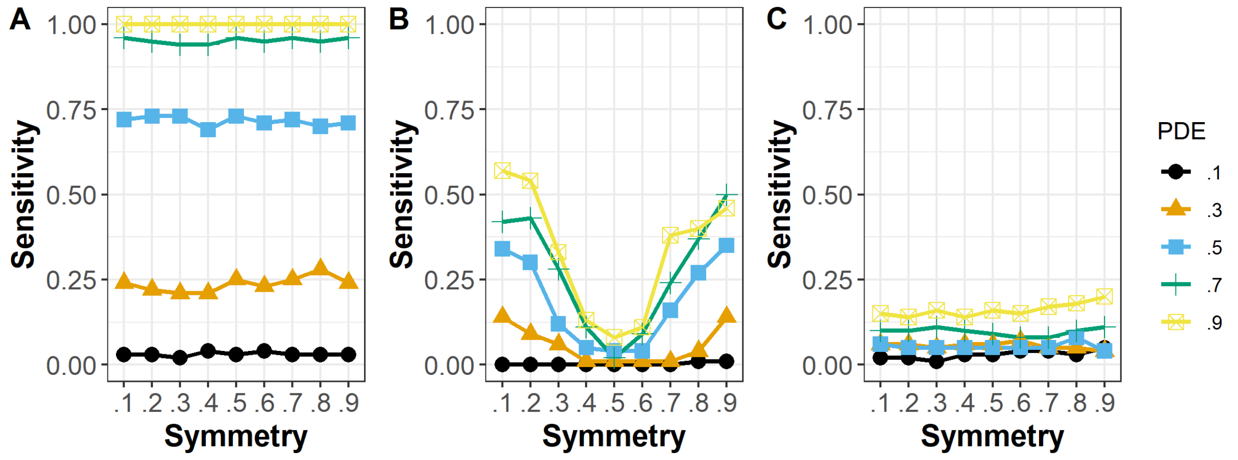

Figure S42: The proportion of inversely changed pathways detected by each method, when the probability of inverse change was 0.3. The results are shown for (A) ECEA, (B) GSEA, and (C) ORA.

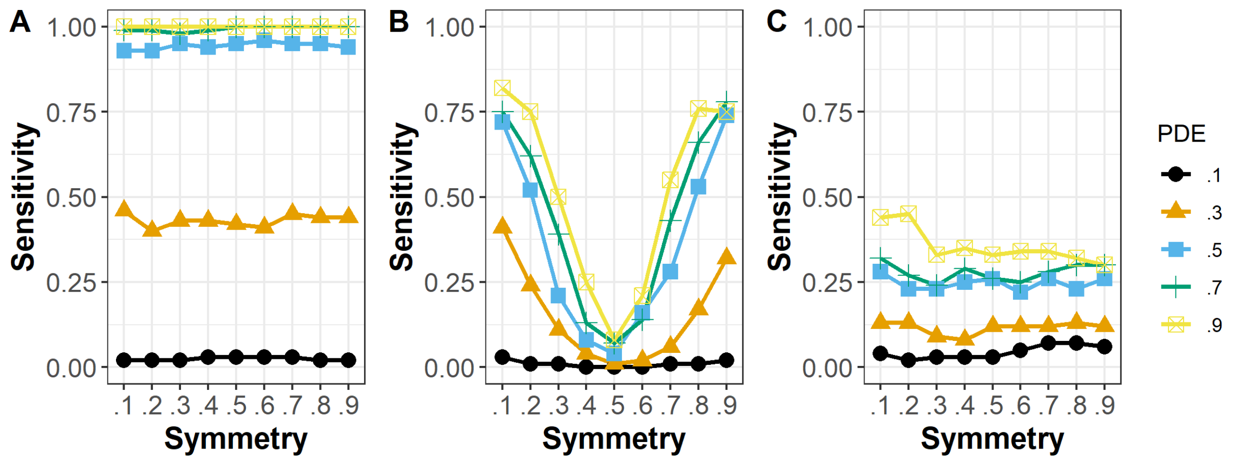

Figure S53: The proportion of inversely changed pathways detected by each method, when the probability of inverse change was 0.4. The results are shown for (A) ECEA, (B) GSEA, and (C) ORA.

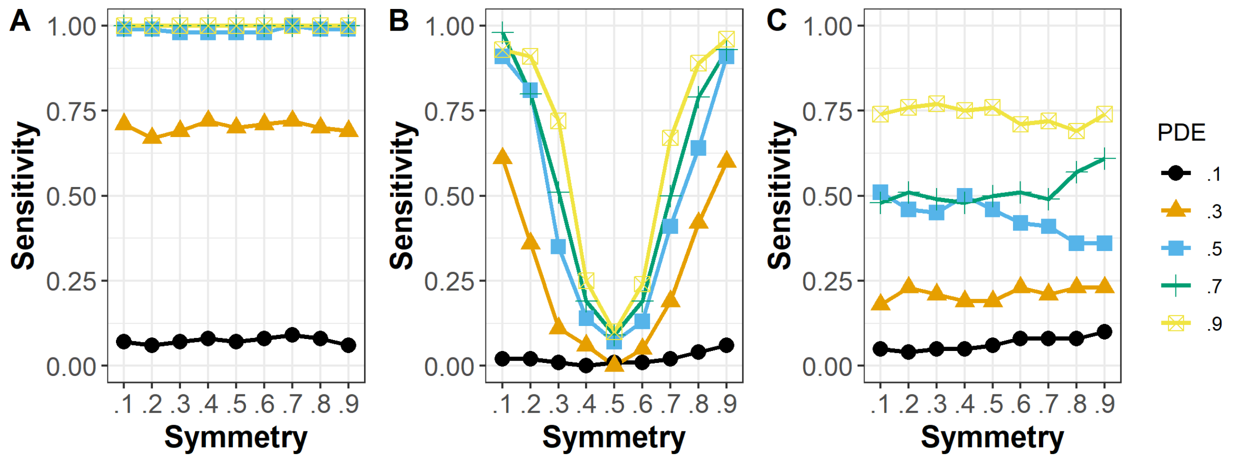

Figure S64: The proportion of inversely changed pathways detected by each method, when the probability of inverse change was 0.5. The results are shown for (A) ECEA, (B) GSEA, and (C) ORA.

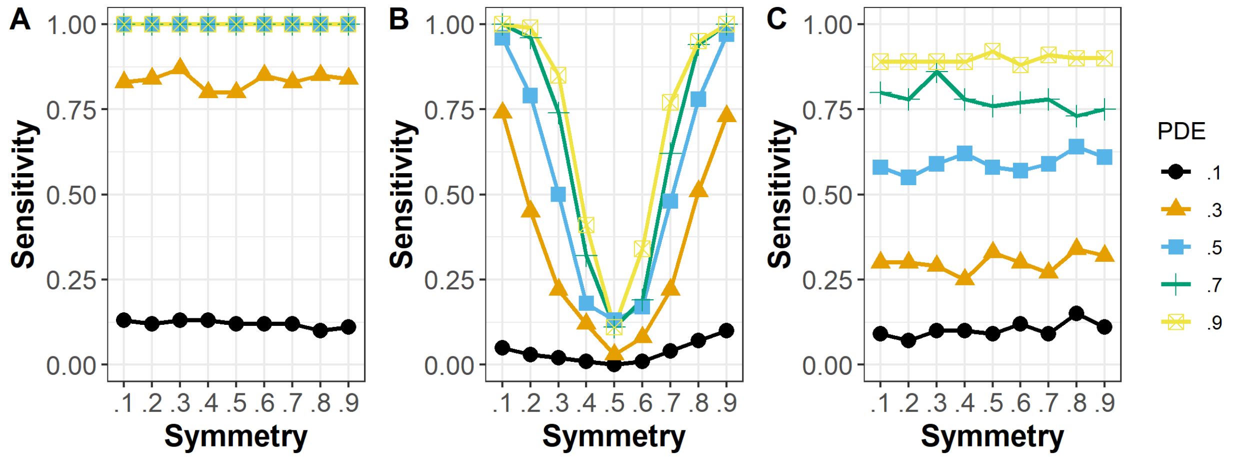

Figure S75: The proportion of inversely changed pathways detected by each method, when the probability of inverse change was 0.6. The results are shown for (A) ECEA, (B) GSEA, and (C) ORA.

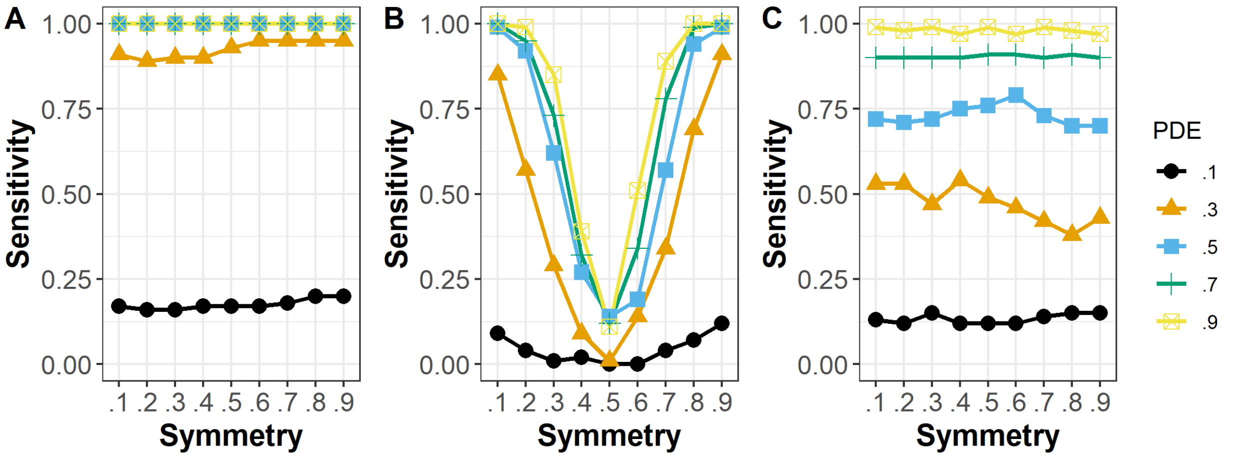

Figure S86: The proportion of inversely changed pathways detected by each method, when the probability of inverse change was 0.7. The results are shown for (A) ECEA, (B) GSEA, and (C) ORA.

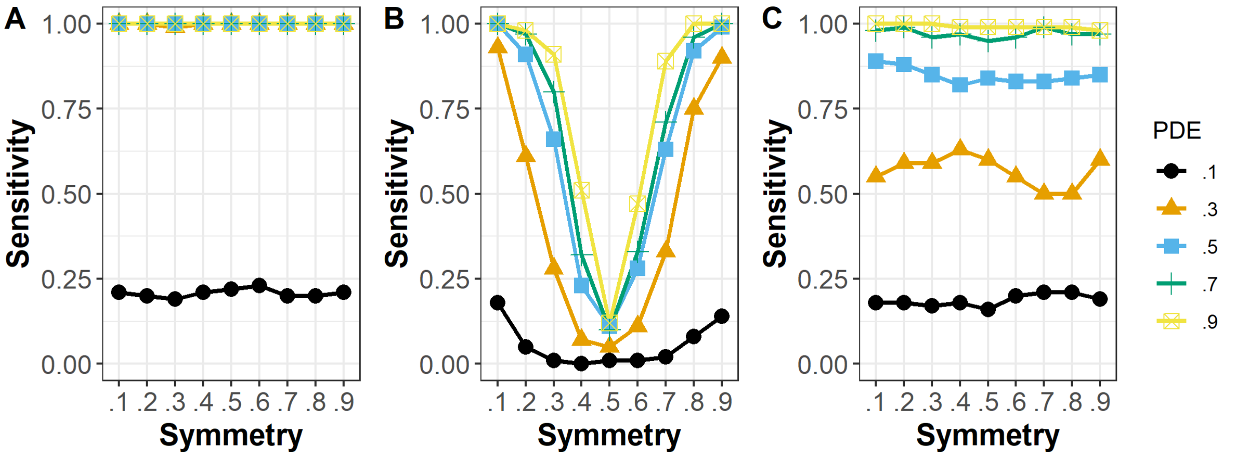

Figure S97: The proportion of inversely changed pathways detected by each method, when the probability of inverse change was 0.8. The results are shown for (A) ECEA, (B) GSEA, and (C) ORA.

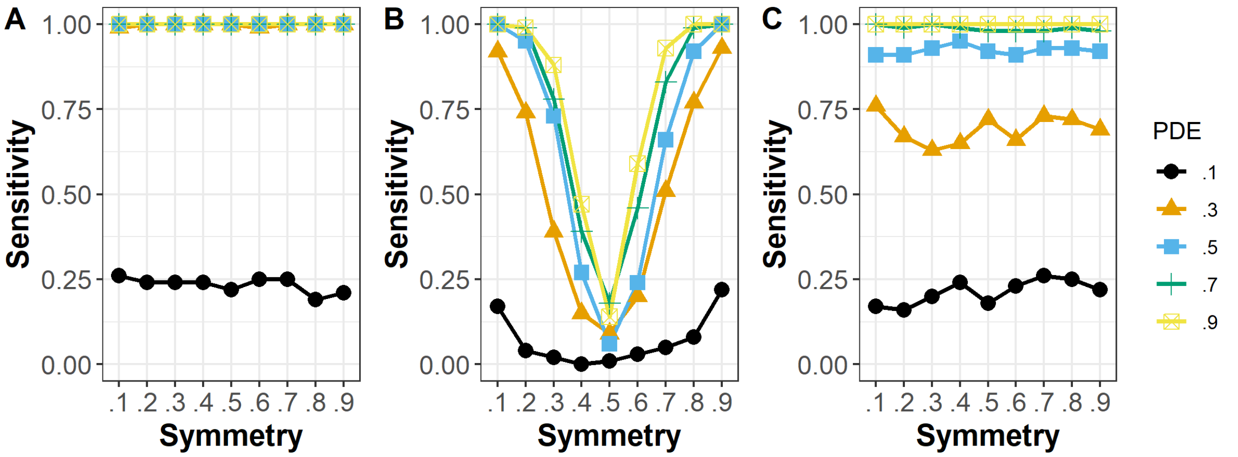

Figure S108: The proportion of inversely changed pathways detected by each method, when the probability of inverse change was 0.9. The results are shown for (A) ECEA, (B) GSEA, and (C) ORA.
